# Alopecia secondary to severe discoid lupus responding to anifrolumab

**DOI:** 10.1097/JW9.0000000000000098

**Published:** 2023-07-25

**Authors:** Shannon Han, James Ferrer, Mohamad Bittar, Allison Jones

**Affiliations:** a College of Medicine, Department of Medicine, The University of Tennessee Health Science Center, Memphis, Tennessee; b Kaplan-Amonette Department of Dermatology, University of Tennessee Health Science Center, Memphis, Tennessee; c Division of Rheumatology, Department of Medicine, The University of Tennessee Health Science Center, Memphis, Tennessee

**Keywords:** alopecia, anifrolumab, discoid lupus erythematosus

**What is known about this subject in regard to women and their families?**

- Anifrolumab is currently approved by food and drug administration for systemic lupus erythematosus.
- Discoid lupus erythematosus (DLE) can cause irreversible scarring and have a significant impact on quality of life.
- Women are affected with discoid lupus in a much greater proportion than men.

**What is new from this article as messages for women and their families?**

- Prior evidence has shown a significant benefit of anifrolumab in patients with systemic lupus erythematosus, but there are minimal peer-reviewed data regarding the efficacy of DLE. Anifrolumab is a therapeutic option that should be considered in patients with refractory or severe DLE. Its benefits include a more favorable side effect profile and greater medication adherence.

## Dear Editors,

### Introduction

Discoid lupus erythematosus (DLE) is a subset of cutaneous lupus that can cause irreversible scarring and profoundly affect the quality of life.^1^ Traditional therapies, including immunosuppressants, retinoids, antimalarials, thalidomide, and lenalidomide, have shown variable success for recalcitrant DLE.^2^ Anifrolumab (ANI), a human monoclonal antibody targeting type I interferon receptor subunit 1, was approved by the Food and Drug Administration (FDA) in 2021 for systemic lupus erythematosus (SLE), as the type 1 interferon pathway has been previously implicated in the pathogenesis of SLE.^3^ The hallmark phase 3 trial TULIP-2 studying ANI therapy in SLE showed a benefit in the reduction of skin lesion severity, with a 50% or more reduction in Cutaneous Lupus Erythematosus Disease Area and Severity Index activity score in 49% of patients treated with ANI versus 25% of patients in the placebo group.^4^ Thus, ANI is a therapeutic option that should be considered in patients with lupus-related skin disease. We describe a patient with refractory DLE treated with ANI.

## Case presentation

**Fig. 1.**
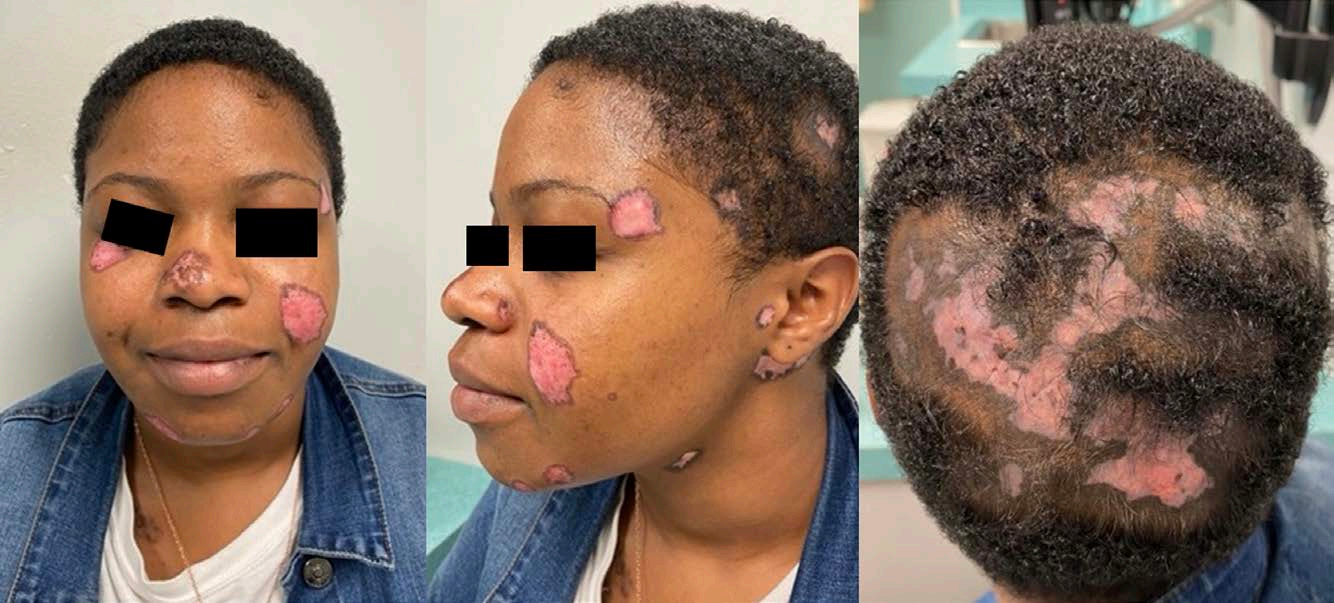
Face and scalp of the patient, before initiation of ANI.

**Fig. 2.**
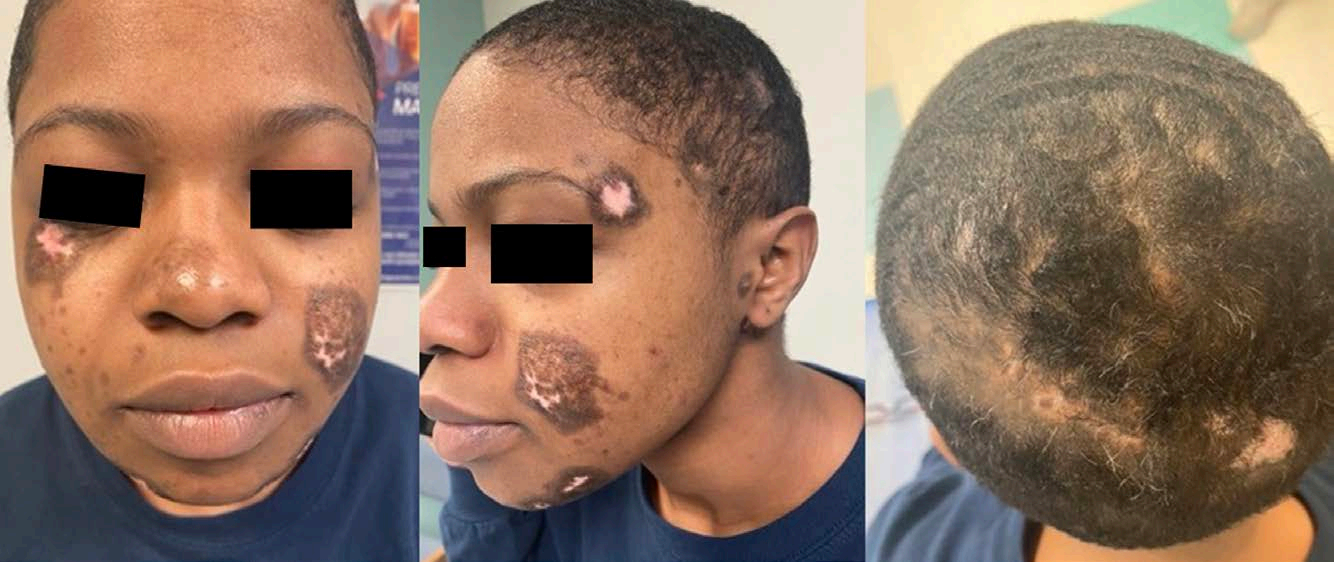
Face and scalp, 7 months after starting ANI.

A 21-year-old woman presented with a 3-year history of discoid rash and alopecia. Initial examination was notable for multiple erythematous atrophic patches with peripheral hyperpigmentation on the scalp, face, ears, and right upper chest within a tattoo, with significant alopecia of the scalp (Fig. 1). She was found to have negative serology for SLE, including anti-nuclear antibody (ANA), rheumatoid factor (RF), cyclic citrullinated peptide (CP), complement (C3 and C4), erythrocyte sedimentation rate (ESR), and C reactive protein (CRP). She had previously tried topical betamethasone with minimal improvement. Treatment with tacrolimus ointment, clobetasol ointment, and prednisone was initiated, with later addition of hydroxychloroquine and azathioprine for an approximate duration of 14 months and 8 months, respectively. Methotrexate was considered, but the patient did not desire contraception. Overall, the patient had minimal response to the standard oral treatment options, and she had difficulty adhering to the treatment regimen. The decision was made to stop azathioprine and to start ANI, despite the lack of data on ANI in pregnancy, with an infusion schedule of 300 mg every 4 weeks. Hydroxychloroquine was continued for an additional 4 months after initiation of ANI but was eventually stopped secondary to prolonged QTc interval. She continued with the prednisone 5 mg and topical treatments. At the 1-month follow-up visit, we observed significant improvement in the appearance and size of the cutaneous lesions, with impressive hair regrowth seen at 7 months (Fig. 2).

## Discussion

Our patient with recalcitrant DLE was transitioned to ANI due to the ineffectiveness of prior therapy and issues with medication compliance. Even after 1 month, the patient showed significant improvement and good tolerability with ANI. The areas of prior DLE on the scalp had almost complete hair regrowth, suggesting ANI halted the inflammatory process before any significant scarring of the hair shaft. Two cases have been previously reported, highlighting similar clinical improvement of cutaneous lesions and patchy alopecia in treatment-resistant DLE.^5^ An additional benefit is the favorable adverse effect profile. Traditional therapies for DLE, including prednisone, hydroxychloroquine, azathioprine, and methotrexate, are associated with significant adverse effects. Morand et al.^4^ found the most common adverse effects observed in ANI were mild and included upper respiratory tract infections. These results showcase the potential for ANI to be a viable and effective therapy for patients with DLE, especially those with refractory or severe DLE. Future studies could investigate the early use of ANI monotherapy to increase medication adherence and forgo the adverse effects associated with traditional therapy.

## Conflicts of interest

None.

## Funding

None.

## Study approval

The study did not require approval.

## Author contributions

SH: Participated in the writing of the original draft. JF: Participated in review and editing of the paper. AJ: Participated in reviewing the paper and approving it for submission, participated in supervision. MB: Participated in reviewing the paper and approving it for submission.

## Patient consent

Informed, written consent was received from all patients for whom photographs are present in the manuscript.
